# Variations in ecosystem service value in response to land use/land cover changes in Central Asia from 1995–2035

**DOI:** 10.7717/peerj.7665

**Published:** 2019-09-12

**Authors:** Jiangyue Li, Hongxing Chen, Chi Zhang, Tao Pan

**Affiliations:** 1 State Key Laboratory of Desert and Oasis Ecology, Xinjiang Institute of Ecology and Geography, Chinese Academy of Sciences, Urumqi, China; 2 University of Chinese Academy of Sciences, Beijing, China; 3 Research Center for Ecology and Environment of Central Asia, Chinese Academy of Sciences, Urumqi, China, Urumqi, China

**Keywords:** Ecosystem services, Value coefficient, LULC change, Central Asia

## Abstract

Acute farmland expansion and rapid urbanization in Central Asia have accelerated land use/land cover changes, which have substantial effects on ecosystem services. However, the spatiotemporal variations in ecosystem service values (ESVs) in Central Asia are not well understood. Here, based on land use products with 300-m resolution for the years 1995, 2005 and 2015 and transfer methodology, we predicted land use and land cover (LULC) for 2025 and 2035 using CA-Markov, assessed changes in ESVs in response to LULC dynamics, and explored the elasticity of the response of ESV to LULC changes. We found significant expansions of cropland (+22.10%) and urban areas (+322.40%) and shrinking of water bodies (−38.43%) and bare land (−9.42%) during 1995–2035. The combined value of ecosystem services of water bodies, cropland, and grassland accounted for over 90% of the total ESVs. Our study showed that cropland ecosystem services value increased by 93.45 billion US$ from 1995 to 2035, which was mainly caused by the expansion of cropland area. However, the area of water bodies decreased sharply during 1995–2035, causing a loss of 64.38 billion US$. Biodiversity, food production and water regulation were major ecosystem service functions, accounting for 80.52% of the total ESVs. Our results demonstrated that effective land-use policies should be made to control farmland expansion and protect water bodies, grassland and forestland for more sustainable ecosystem services.

## Introduction

Ecosystem services (ES) refer to the direct and indirect benefits that people obtain from ecosystems (Costanza et al., 1997), including provisioning services (food and raw material), regulating services (water regulation, climate regulation and gas regulation), supporting services (soil formation, waste treatment and biodiversity) and cultural services (recreation, cultural and tourism) (Hassan, Scholes & Ash, 2005). Quantifying the benefits obtained from ecosystems can be achieved through evaluation of ecosystem services values (ESV) in monetary units (Costanza et al., 2014; Metzger, Rounsevell & Acosta, 2006). The measurement of ESV in monetary units is an important step to improve incentives and obtain expenditures needed for their conservation and sustainable use (e.g., systems of Payments for Ecological Services) (Farley & Costanza, 2010). In addition, these values can help policy makers make optimal decisions on the rational allocation of resources and provide useful information for understanding user interests and the relative value of current ES (Farley, 2008).

For the evaluation of ESV in monetary units, four methods were found in Talberth (2015), including stated preference method, revealed preference method, cost-based method and benefit transfer method. Among the various methods of regional or global ES assessment, benefit transfer method (BTM) has been widely used because of its feasibility and simplicity (Costanza et al., 1997; Costanza et al., 2014; DeGroot et al., 2012). This method suggests that the value of each ecosystem service functions uses specific value for a particular land cover, obtaining from single or multiple case studies (DeGroot et al., 2012). Most notably, the global biosphere was first classified into 16 sub-ecosystems and 17 ecosystem service functions, and the value of each ES was evaluated by BTM (Costanza et al., 1997). However, some researchers have severely criticized their findings (e.g., Serafy, 1998; Wilson & Howarth, 2002) due to the limitations and uncertainties in their local use (Kindu et al., 2016; Li et al., 2007). Recently, the evaluation has been updated based on more than 300 case studies worldwide (Costanza et al., 2014; DeGroot et al., 2012). Costanza et al. (2014) claimed that the models and basic data they use in their evaluation can be applied at various scales to evaluate dynamic changes of ESV.

Land use and land cover (LULC) changes alter structures and functions of ecosystems and influences the supply of ecosystem services (Hu, Liu & Min, 2008; Kreuter et al., 2001; Yirsaw et al., 2017). Excessive utilization of land resources may lead to severe degradation or loss of local or regional ES (Collin & Melloul, 2001). Recent research has shown that cropland conversion, urbanization and deforestation have led to the loss of carbon sequestration, reduction in biodiversity, decline in water quality, and land degradation, and, resulting in a significant decline in ESV (Maitre et al., 2007; Polasky et al., 2011). Numerous studies have evaluated the impact of LULC dynamics on ES worldwide by utilizing the valuation coefficients of Costanza et al. (1997) and Costanza et al. (2014). For example, Arowolo et al. (2018) studied landuse change and its impacts on ESV in Nigeria. Yi et al. (2017) assessed the effects of land-use change on ES in the San Antonio River Basin, Texas. These studies have provided valuable references for land-use policy makers.

Situated in the center of the Eurasian continent, Central Asia dryland has experienced complex LULC changes since the collapse of the Soviet Union in the early 1990s (Behnke & Mortimore, 2016; Hamidov, Helming & Balla, 2016). These changes have had profound effects on the fragile ecological environment in Central Asia. For example, animal husbandry heavily relies on the grassland resources in the study area as a major food supply (Han et al., 2016; Huang, Luo & Han, 2018). The vast pastures of Central Asia constitute the largest continuous grazing area in the world (Gintzburger et al., 2003). Furthermore, grasslands provide other important ecosystem services, such as carbon sequestration, climate and gas regulation, soil and water conservation, and biodiversity (Eichelmann et al., 2016; Grace, 2010; Huang, Luo & Lv, 2017). In recent years, water-stressed grassland ecosystems have been frequently disturbed by human activities (e.g., grazing and reclamation) and climate change, which has led to the decline of grassland ecosystem service quality (Chen et al., 2018; Han et al., 2018; Hobbs & Norton, 1996; Tanentzap & Coomes, 2012). Additionally, the Syr Darya and Amu Darya Rivers are essential sources of water used for agriculture in the study area (Kulmatov et al., 2017). In the Amu Darya River Basin in the Khorezm Province of Uzbekistan and the Fergana Valley, the efficiency of the irrigation and drainage systems from agricultural fields is extremely low (Awan et al., 2016; Karimov et al., 2014). Excessive irrigation not only leads to the waste of large amounts of water resources in the Amu Darya Delta but also results in fertilizer loss, soil salinization and salt storms (Devkota et al., 2015). Because of irrational land use and improper management, there is serious soil erosion, desertification and extensive land degradation in some basins. Therefore, it is urgent to evaluate the impacts of human disturbances on ES in Central Asia. Such a study is important for ecological monitoring, sustainability management and disturbance regulation in this fragile dryland ecosystem. Except for a few qualitative estimates on ES changes in response to LULC changes (Chen, Bai & Li, 2013; Chi et al., 2016), to date, there have been no quantitative assessments of ESV in Central Asia.

Therefore, the aim of this study is as follows: (1) to estimate and project the LULC changes in Central Asia during the period 1995–2035; (2) to evaluate changes in ESV in response to LULC changes; and (3) to explore the elasticity of the response of ESV to LULC changes by 50% adjustment of value coefficients. Our findings could provide policy makers with important references for ecological environmental protection and the sustainable development of Central Asia.

## Materials & Methods

### Study area

**Figure 1:**
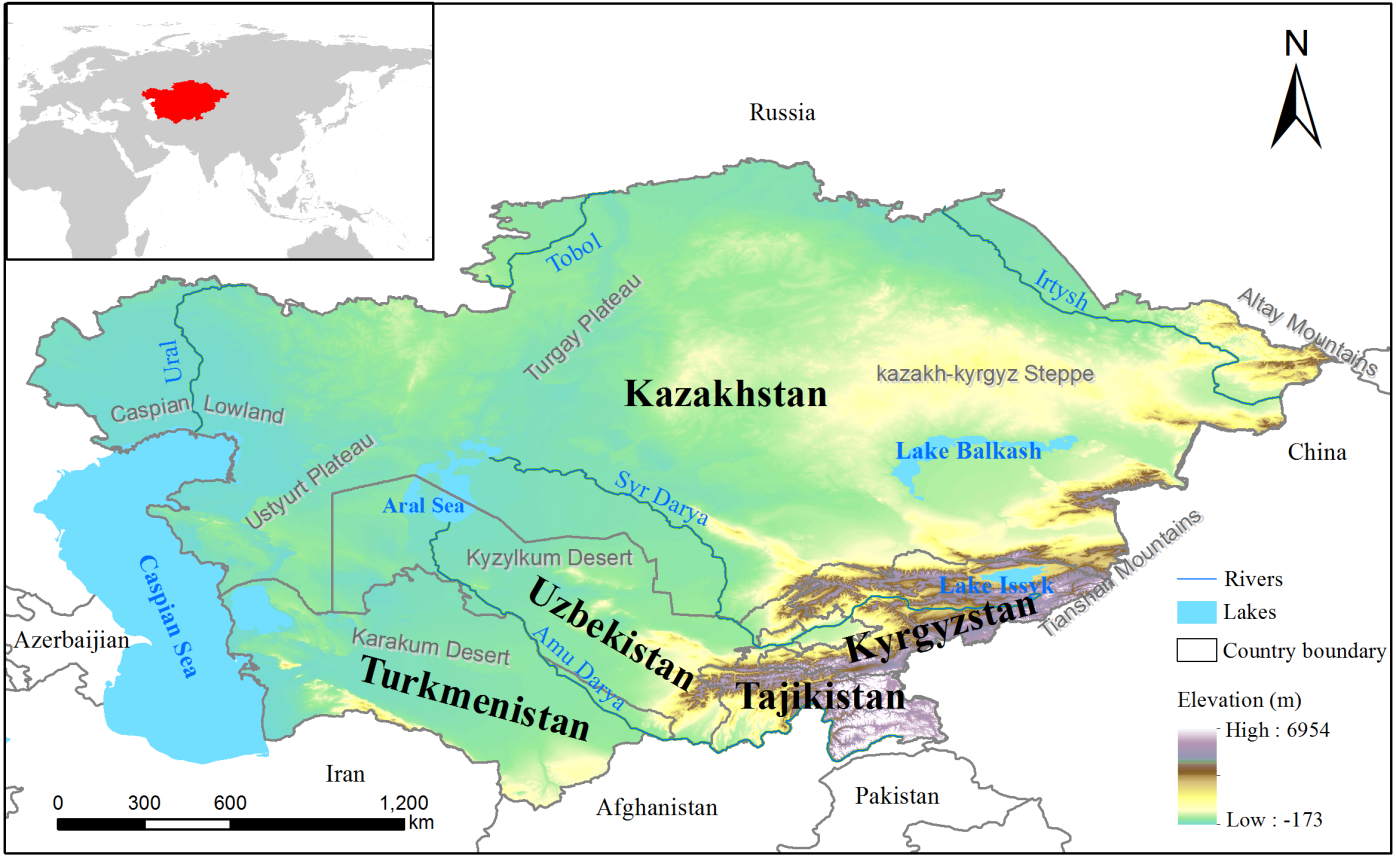
Map showing the location of Central Asia.

Our study area covered five Central Asian countries (from 35°08′N, 55°25′N to 46°28′E, 87°29′E), including Tajikistan, Kyrgyzstan, Turkmenistan, Uzbekistan and Kazakhstan (Fig. 1). With a land area of 4 million km^2^, Central Asia extends from Russia in the north to Afghanistan in the south and from the Caspian Sea in the west to western borders of China in the east (Ozturk et al., 2017). The terrain gradually changes from the western Caspian lowlands to the Altai Mountains, Tianshan Mountains and Pamirs (Beurs et al., 2015). Because this area is located far from the ocean in the hinterland of the Eurasian continent, it has a distinctive continental arid and semiarid climate with low precipitation and intensive evaporation (Lioubimtseva & Henebry, 2009). In recent decades, potential evapotranspiration has increased in Central Asia, especially in the Aral Sea region and western Kazakhstan, with an annual increase of 7.42 mm/year (Jiang et al., 2019). Central Asia has a heterogeneous landscape, including diverse land-cover types such as temperate deserts (e.g., Kyzylkum, Karakum Desert), forests, lakes (e.g., the Aral Sea, Balkhash Lake) and vast grasslands. Major transboundary rivers, such as the Syr Darya, the Amu Darya, the Irtysh River, and the Ili River, are critical water sources for regional ecosystems and agriculture (Zhou et al., 2019). With the large-scale development of irrigated agriculture in the Aral Sea Basin in the early 1960s, the irrigated cultivated land area increased by 60%, and the planted cotton area doubled between the 1960s and the 1990s. Now, irrigated land accounts for almost half of the total cultivated land area (Suo et al., 2009).

### Data collection and land use classification

Multiscale satellite observations were used to characterize LULC change (Song, 2018). In a preliminary study, we compared the multiple LULC datasets for the study area, including the GLC2000 (Corresponding & Belward, 2005), the GlobCover 2009 (Arino et al., 2008), MODIS land cover (Friedl et al., 2010), and the newly released annual European Space Agency Climate Change Initiative land cover maps (CCI-LC) (http://maps.elie.ucl.ac.be/CCI/viewer) (Radoux et al., 2013). The comparison showed that the CCI-LC has the highest spatial resolution and has better accuracy in the study area (Hartley et al., 2017; Wei et al., 2015). This product was developed using the GlobCover unsupervised classification chain and combined a variety of earth observation products based on ESA’s GlobCover products. Unlike many remote sensing products based on single-sensor methods, this dataset is generated using multiple sensors, such as the Advanced Very High Resolution Radiometer (AVHRR), System Probatoire d’Observation de la Terre Vegetation (SPOT-VGT), and PROBA-V. To validate the data, Defourny et al. (2016) compared the CCI LC product for the 2010 with the certain and homogeneous points of the GlobCover 2009, with a total accuracy of 73.2% (Georgievski & Hagemann, 2019). More high-accuracy land classifications were found: water bodies (92%–96%), irrigated cropland (89%–92%), rainfed irrigated cropland (89%–83%), bare land (89%–88%) and urban areas (88%–86%) (Defourny et al., 2016). However, natural vegetation and sparse vegetation have low user accuracy values, but all errors relate to confusion between these and other LULC classes, which very much limits the impact of the low values. Details about the CCI-LC dataset, including its accuracy and the confusion matrix, can be found in the study by Defourny et al. (2016). To match Costanza’s biomes, plant functional types were classified into seven major LULC types, including cropland, forestland, grassland, wetland, urban areas, bare land and water bodies (Table S1).

### Model projection of future LULC change

Future LULC change from 2015 to 2035 was projected by combining a Geographic Information System (GIS) with a CA-Markov model, which is a robust approach for simulation of the spatial—temporal change patterns of LULC (e.g., Fu, Wang & Yang, 2018; Hartley et al., 2017; Muller & Middleton, 1994). The CA-Markov model merges cellular automata (CA) and Markov chain (Ji et al., 2009). The Markov chain is constructed based on the probability of change matrices for LUCC from *t* to *t* + 1:

\begin{eqnarray*}{s}_{ \left( t+1 \right) }={s}_{ \left( t \right) }\times A \end{eqnarray*}

where ${s}_{ \left( t+1 \right) }$ is the state probability of any time, and ${s}_{ \left( t \right) }$ is the initial state probability. *A* is the transition probability matrix, and the formula is as follows:

\begin{eqnarray*}A= \left[ \begin{array}{@{}cccc@{}} \displaystyle {A}_{11}&\displaystyle {A}_{12}&\displaystyle \ldots &\displaystyle {A}_{1n}\\ \displaystyle {A}_{21}&\displaystyle {A}_{22}&\displaystyle \ldots &\displaystyle {A}_{2n}\\ \displaystyle \ldots &\displaystyle \ldots &\displaystyle \ldots &\displaystyle \ldots \\ \displaystyle {A}_{n1}&\displaystyle {A}_{n2}&\displaystyle \ldots &\displaystyle {A}_{nn} \end{array} \right] \end{eqnarray*}

where *A*_*ij*_ is the sum of areas from the *i*th land cover category to the *j*th category from the initial period to forecast periods, and *n* is the number of LULC categories. We accomplished this process by using the MARKOV module in the IDRISI, a software that integrates GIS and image processing functions.

The CA model is used to predict the space–time dynamic change pattern using a transition map of the LULC (Yang, Zheng & Lv, 2012), and the model can be defined as follows (Wang, Zheng & Zang, 2012):

\begin{eqnarray*}{S}_{ \left( t,t+1 \right) }=f \left( {S}_{ \left( t \right) },N \right) \end{eqnarray*}

where *S* is a set of cellular states, *N* is the cellular field, *t* and *t* + 1 represent different time periods, and *f* is the local transition rule of the cell.

To ensure the reliability of the simulation results, we used the kappa index to test the consistency level of the simulated and observed land cover maps (Mitsova, Shuster & Wang, 2011):

\begin{eqnarray*}kappa= \frac{{p}_{0}-{p}_{c}}{1-{p}_{c}} \end{eqnarray*}

where *kappa* is the index of simulation accuracy, *p*_*c*_ is the expected simulation accuracy in a random state, and *p*_0_ is the actual simulation accuracy.

By incorporating the advantages of these two methods, the CA–Markov model is able to accomplish a better simulation of LULC changes both in quantity and space (Yang et al., 2014). In this study, the transition probability matrix was performed for the time period between 1995 and 2005 to predict the LULC map of 2015, which would be used to verify model accuracy. Then, using the 2015 classified map as the LULC baseline and the 2005 and 2015 maps for the transition probability matrix, we predicted the 2025 and 2035 LULC maps with the CA-Markov model.

**Table 1:** The value coefficient of ecosystem services on seven LULC categories in Central Asia. (US$ha ^1^ yr ^1^).

Service type	Sub-type	Cropland	Forestland	Grassland	Wetland	Urban	Bare land	Waterbodies
Provisioning	Food production	2,323	299	1,192	614	0	0	106
	Raw material	219	181	54	539	0	0	0
Regulating	Gas regulation	0	0	9	0	0	0	0
	Climate regulation	411	152	40	3,474	905	0	0
	Water regulation	400	191	63	6,014	16	0	9,322
Supporting	Soil-formation and retention	639	107	46	4,320	0	0	0
	Waste-treatment	397	120	75	3,015	0	0	918
	Biodiversity	1,096	1,097	2,494	3,502	0	0	0
Culture	Recreation, cultural and tourism	82	990	193	4,203	5,740	0	2,166
Total ecosystem value		5,567	3,137	4,166	25,681	6,661	0	12,512

### Assessment of ecosystem service values

In this study, ESV were estimated based on benefit transfer method (BTM) proposed by Costanza et al. (1997). Nine ecosystem service functions generated from Xie et al. (2008), derived from the 17 ecosystem services listed by Costanza et al. (1997), were selected in this assessment (Sannigrahi et al., 2018). Cultivated land, temperate forest, grassland/shrubland, swamps/floodplains, urban areas, and desert/tundra/ice/bare-rocks in Central Asia were matched to cropland, forests, grassland, wetland, urban and bare land in Costanza et al.’s (2014) model, respectively (Table S1). The biomes that we used as proxies for the 7 LULC categories are not perfectly matched to those of Costanza et al.’s (1997) ESV model in some cases (Table S1), but they are closely related (Kreuter et al., 2001). The equivalent value coefficient of each ES updated by Costanza et al. (2014) was used to calculate ecosystem services values (Table 1). The formula is as follows:

\begin{eqnarray*}& & ES{V}_{k}={\Sigma }_{f}{A}_{k}\times V{C}_{kf} \end{eqnarray*}

\begin{eqnarray*}& & ES{V}_{f}={\Sigma }_{k}{A}_{k}\times V{C}_{kf} \end{eqnarray*}

\begin{eqnarray*}& & ESV={\Sigma }_{f}{\Sigma }_{k}{A}_{k}\times V{C}_{kf} \end{eqnarray*}

where *ESV*_*k*_ refers to the ecosystem service value of LULC type ‘*k*’, *A*_*k*_ represents the area (ha) for LULC category ‘*k*’, and *VC*_*fk*_ is the value coefficient (US $/ha/year) of function  *f* for the LULC type ‘*k*’ (Kreuter et al., 2001). *ESV*_*f*_ is the ecosystem service value of service function *f*, and *ESV* is the total ecosystem service value. We use the following formula to evaluate changes in ESV:

\begin{eqnarray*}ES{V}_{cr}= \frac{ES{V}_{t2}-ES{V}_{t1}}{ES{V}_{t1}} \times 100\text{%}. \end{eqnarray*}

In this expression, *ESV*_*cr*_ refers to the change rate of ESV from the initial year to the final year, and *ESV*_*t*1_ and *ESV*_*t*2_ represent the total *ESV* at the start and end of the study period, respectively.

### Elasticity for the response of ESV to LULC change

The biomes that we used as proxies for the 7 LULC categories are not perfectly matched to Costanza et al.’s (1997) ESV model in some cases (Table S1), which results in uncertainties in the assessment of the ESV. Thus, we used sensitivity analysis to evaluate the changes in ESV in response to 50% adjustments of the ESV coefficients for each LULC type (Kindu et al., 2016). The standard economic concept of elasticity was used to calculate the coefficient of sensitivity (CS) using the following formula (Kreuter et al., 2001):

\begin{eqnarray*}CS= \frac{(ES{V}_{j}-ES{V}_{i})/ES{V}_{i}}{(ES{V}_{jk}-ES{V}_{ik})/ES{V}_{ik}} \end{eqnarray*}

where *ESV* is the estimated total value of ecosystem services, *VC* is the value coefficient, and ‘*i*’, ‘*j*’ and ‘*k*’ represent the initial, adjusted values, and LULC categories, respectively. If *CS* >1, then the estimated ESV is elastic with respect to that coefficient; if *CS* ≤1, the estimated ESV is inelastic. Thus, when *CS* <1, even if the accuracy of *VC* values used as proxy biomes is low, the results of estimation of *ESV* are credible (Kreuter et al., 2001).

## Results

### Analysis of LULC dynamics

Combining the GIS technology with the CA-Markov model, we used the LULC base map from 2005 and transition probabilities from 1995 to 2005 to simulate the LULC for 2015. The kappa statistic of 0.93 indicates that there is good consistency with the actual value of the LULC types and the predicted results for the base year. Then, the future LULC in 2025 was predicted with the CA-Markov model in IDRISI using the LULC base map from 2015 and the transition probabilities from 2005 to 2015. Following the above process, the future LULC in 2035 was predicted.

**Figure 2:**
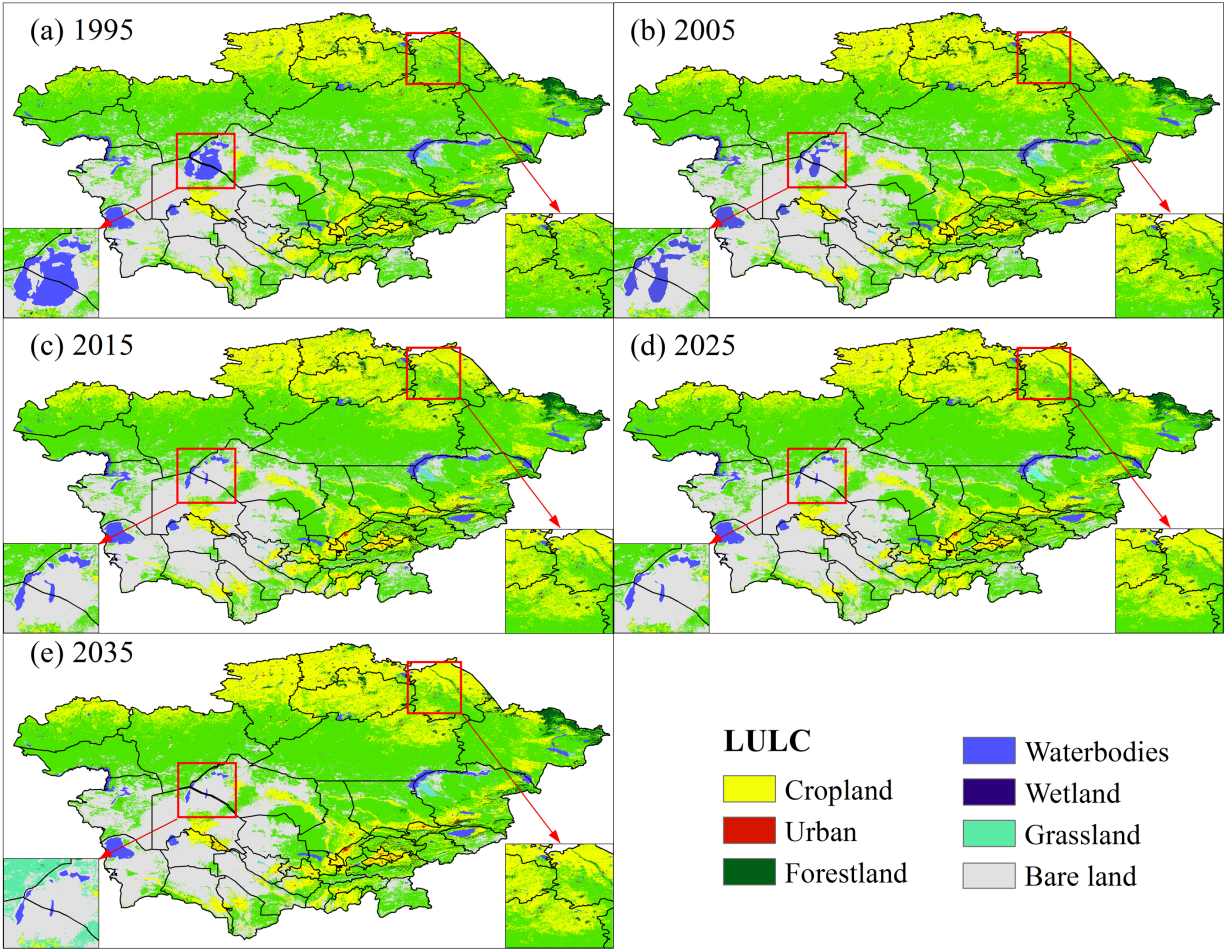
Spatial distribution of LULC in Central Asia. (A) 1995, (B) 2005, (C) 2015, (D) 2025, (E) 2035.

**Figure 3:**
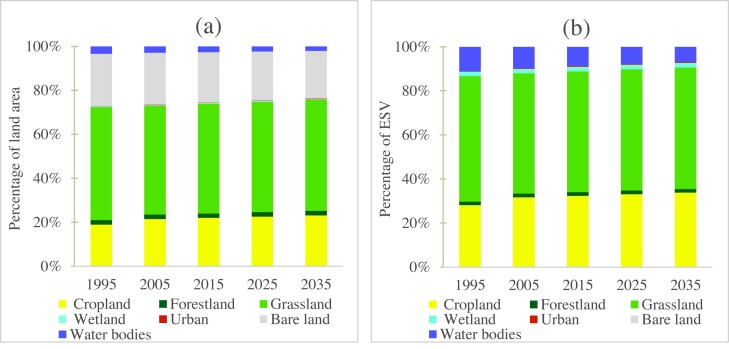
(A) The percentage of land use area and (B) the percentage of ecosystem service value of different land use types.

Figure 2 presents the patterns of spatial distribution of LULC in Central Asia from 1995 to 2035, and Table 2 shows the magnitude of changes for the same periods. In 1995, grassland occupied approximately 51.39% of the study area, followed by bare land and cropland, which occupied 23.90% and 18.98% of the study area, respectively (Fig. 3). During 1995–2015, cropland and urban area increased substantially. The cropland expanded at a rate of 0.76% per annum, increasing 1,219.56  × 10^4^ ha by the year 2015 (Table 2). Rapid urbanization also caused the proportion of urban build-up to increase from 27.57 × 10^4^ ha in 1995 to 60.21  × 10^4^ ha in 2005 and then to 89.19  × 10^4^ ha in 2015, with an average growth rate of 10.64% per year (Table 2). Cropland and urban areas were expected to continue to increase in the 2025 and 2035 periods. However, the coverage of bare land, mainly concentrated in the Kyzylkum Desert and Karakum Desert, decreased from 23.90% to 22.95% during 1995–2015 and was projected to further decrease to 21.65% by 2035.

Wetlands and water bodies only account for 3% of the total study area. Wetlands increased from 121.14 ×10^4^ ha in 1995 to 125.25 ×10^4^ ha in 2015 and 128. 41 ×10^4^ ha in 2035 (Table 2), mainly near Balkhash Lake (Fig. 2). The area of water bodies decreased 292.59  × 10^4^ ha from 1995 to 2015 and is expected to further decrease 221.90 × 10^4^ ha by 2035. Among water bodies in Central Asia, the Aral Sea has shrunk most dramatically (Fig. 2).

**Table 2:** Area changes of LULC in Central Asia from 1995 to 2035.

	LULC	Cropland	Forestland	Grassland	Wetland	Urban	Bare land	Water bodies	Total
area (*10^4^ ha)	1995	7,595.03	800.90	2,0560.52	121.14	27.57	9,563.26	1,342.06	4,0010.48
	2005	8,591.77	801.05	19,810.87	121.69	60.21	9,449.80	1,175.08	4,0010.48
	2015	8,814.59	800.48	19,948.14	125.25	89.19	9,183.36	1,049.48	4,0010.48
	2025	9,051.58	799.49	20,077.26	125.25	90.82	8,926.89	939.17	4,0010.48
	2035	9,273.75	798.42	20,203.47	128.41	116.47	8,662.38	827.58	4,0010.48
changes (%)	1995–2015	16.06	−0.05	−2.98	3.39	223.45	−3.97	−21.80	–
	2015–2035	5.21	−0.26	1.28	2.52	30.59	−5.67	−0.21	–
	1995–2035	22.10	−0.31	−1.74	6.00	322.40	−9.42	−38.34	–

### Changes in total ecosystem services values

According to our estimation, the total ESV in Central Asia was approximately 1,505.31 billion US$ in 1995 (Table 3). Grassland had the highest contribution of 56.90%, followed by cropland and water bodies (28.09% and 11.15%, respectively) (Fig. 3). Due to LULC change, the regional ESV increased 5.68 billion US$ during 1995–2005, mainly due to the increased ESV in cropland and urban build-up, which overcompensated the ESV loss in grassland and water bodies. The regional ESV further increased 5.23 billion US$ from 2005–2015. Overall, the ESV in Central Asia increased 10.91 billion US$ during 1995–2015. It is noteworthy that the proportion of water bodies decreased sharply by 21.80% from 1995–2015, causing a loss of 36.61 billion US$. These trends will continue to occur in 2025 and 2035 (Table 3).

**Table 3:** Ecosystem service value of Central Asia from 1995 to 2035.

LULC	ESV (billion US$)					Changes (%)		
	1995	2005	2015	2025	2035	1995–2015	2015–2035	1995–2035
Cropland	422.79	478.27	490.68	503.87	516.24	16.06	5.21	22.10
Forestland	25.12	25.13	25.11	25.08	25.05	−0.05	−0.26	−0.31
Grassland	856.54	825.31	831.02	836.40	841.66	−2.98	1.28	−1.74
Wetland	31.11	31.25	32.17	32.47	32.98	3.39	2.52	6.00
Urban	1.84	4.01	5.94	6.45	7.76	223.41	30.59	322.33
Bare land	0.00	0.00	0.00	0.00	0.00	0.00	0.00	0.00
Water bodies	167.92	147.02	131.31	117.51	103.54	−21.80	−21.14	−38.34
Total	1,505.31	1,510.99	1,516.23	1,521.78	1,527.22	0.73	0.73	1.46

**Figure 4:**
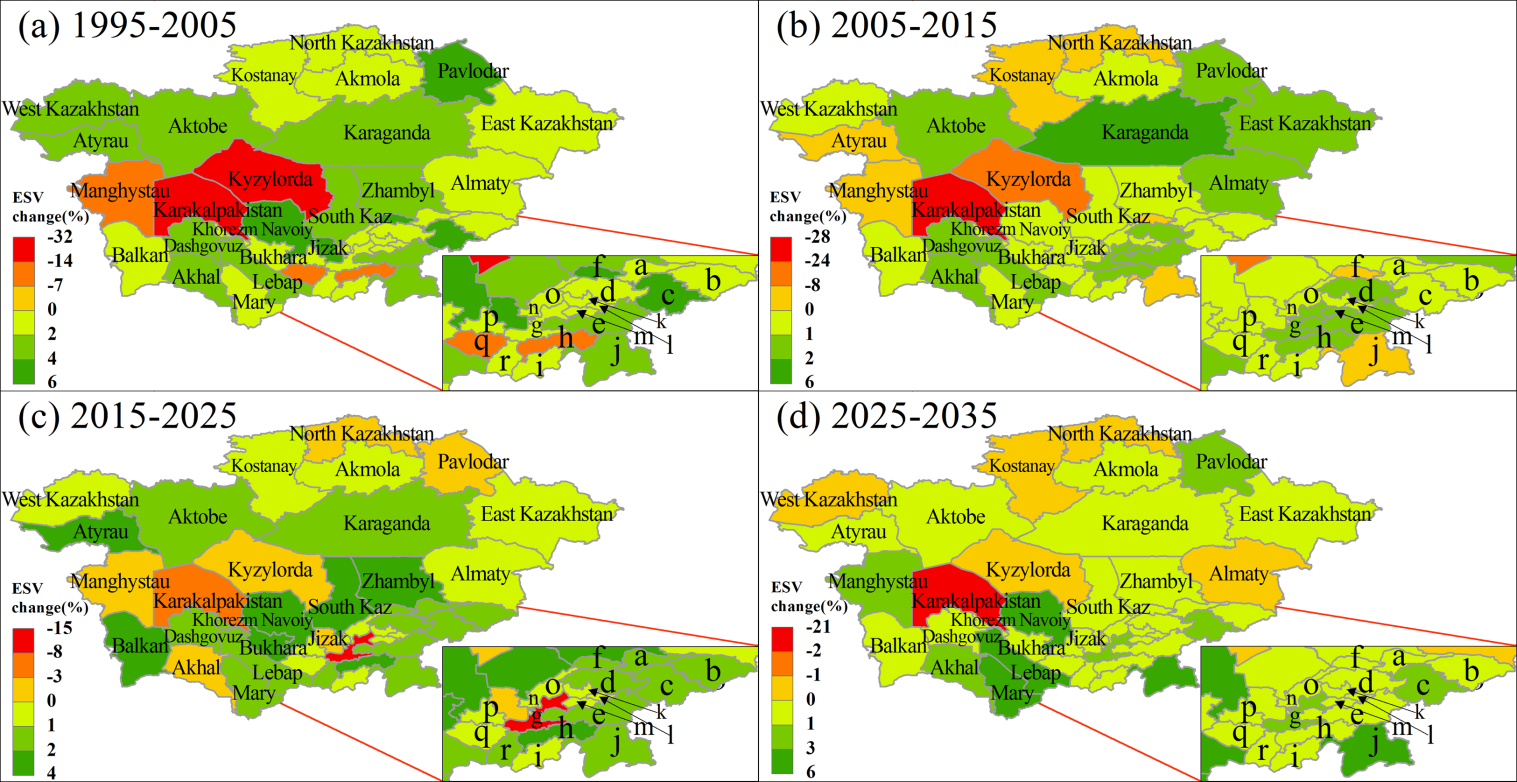
Ecosystem service value change rate (%) from 1995 to 2005 (A), 2005 to 2015 (B), 2015 to 2025 (C) and 2025 to 2035 (D). The state names of a–r can be found in Table S2.

We further analyzed the ESV for administrative units in 1995 (Fig. S1). The highest ESV was found in the Karaganda state (174.09 billion US$), followed by East Kazakhstan (131.69 billion US$), Aktobe (121.09 billion US$) and Almaty (116.70 billion US$) states. The ESV for these administrative units were mainly contributed by grassland in Karaganda (86.13%), East Kazakhstan (67.14.8%) and Aktobe (86.39%). Andijon had the lowest ESV of 2.17 billion US$, 93.73% of which was contributed by cropland. In addition, we calculated the ESV change rates during 1995–2015, 2005–2015, 2015–2025 and 2025–2035 for the administrative units (Fig. 4). From 1995–2005, the ESV of Karakalpakistan, Uzbekistan declined 31.08%, with a total loss of 17.36 billion US$ (Fig. 4A). The ESV in Kyzylorda also decreased substantially, which was mainly caused by shrinking of water bodies. In contrast, the ESV in Pavlodar and Karaganda increased substantially, mainly due to the increased cropland ESV. During 2005–2015, the ESV in Karakalpakistan, Kyzylorda and Manghystau further decreased (Fig. 4B), with the highest ESV loss found in Karakalpakistan (−24.77%), followed by Kyzylorda (−8.83%) and Manghystau (−1.05%). In contrast, Karaganda had the highest increase rate of ESV (5.72%), followed by Aktobe (1.41%) and Almaty (1.25%). It is worth noting that the trend of decreasing ESV will continue to occur in Karakalpakistan and Kyzylorda from 2015 to 2035 (Figs. 4C and 4D).

### Changes in values of ecosystem service functions

**Figure 5:**
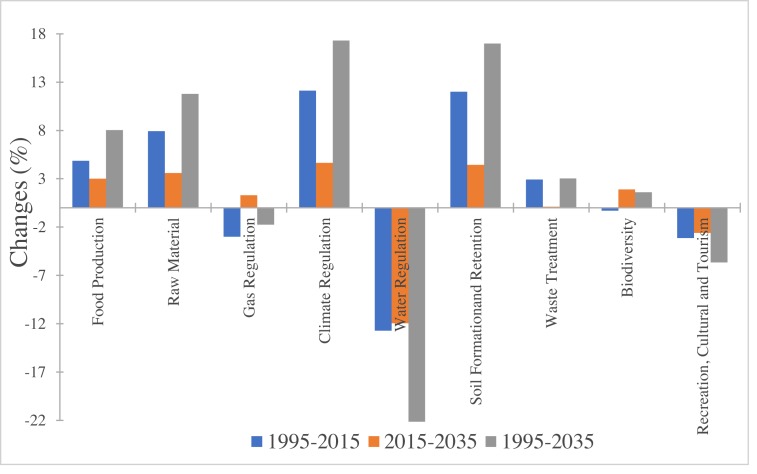
Change rate of ecosystem service function in Central Asia from 1995 to 2035.

Table 4 shows the changes in individual ecosystem functions (*ESV*_*f*_). The most important *ESV*_*f*_ in Central Asia were biodiversity, food production and water regulation, which contributed to 40.44%, 28.30% and 11.78% of the total ESV in 1995, respectively, 40.03%, 29.47% and 10.21% of the total ESV in 2015, and 40.51%, 30.14% and 8.93% of the total ESV in 2035. Most of the *ESV*_*f*_ decreased during 1995–2015 except for food production, raw materials, climate regulation, soil formation and waste treatment, which increased by 4.87%, 7.92%, 12.11%, 12.01%, and 2.91%, respectively (Fig. 5). It is noteworthy that the ESV of water regulation declined more rapidly than other ecosystem services (−12.70%), followed by gas regulation (−3.00%), cultural and tourism (−3.14%) and biodiversity (−0.29%). However, most of the ESV_f_ were projected to increase from 2015–2035 (Fig. 5). Only the ESV of water regulation and cultural service/tourism were expected to decrease in the future.

### Ecosystem sensitivity analysis

In the observed (1995–2015) and projected (2025–2035) study periods, CS for grassland was the highest (0.55) due to the high service value coefficient and large grassland area (Table 5). Meanwhile, CS for cropland increased from 0.28 in 1995 to 0.32 in 2015 and to 0.34 in 2035. Compared with grassland and cropland, the CS (0.02) for forestland was relatively stable. The CS for water bodies decreased from 0.11 in 1995 to 0.09 in 2015 and to 0.07 in 2035. In this study, all CS were far less than “1”, indicating that the total estimated ecosystem values are inelastic with respect to the ecosystem value coefficients.

**Table 4:** Estimated values for different ecosystem functions in Central Asia in 1995–2035.

Service type	Sub-type	Ecosystem service value (billion US$)				
		1995	2005	2015	2025	2035
Provisioning	Food production	426.08	440.12	446.82	453.74	460.31
	Raw material	29.85	31.64	32.22	32.80	33.37
Regulating	Gas regulation	1.87	1.80	1.81	1.82	1.83
	Climate regulation	45.09	49.20	50.56	51.59	52.90
	Water regulation	177.36	165.34	154.83	145.58	136.33
Supporting	Soil formationand retention	63.98	70.02	71.66	73.23	74.84
	Waste treatment	62.49	64.37	64.31	64.33	64.38
	Biodiversity	609.01	601.26	607.24	613.05	618.73
Culture	Recreation, cultural and tourism	89.59	87.25	86.78	84.92	84.52
Total		1,505.32	1,510.99	1,516.23	1,521.78	1,527.23

**Table 5:** Percentage change in estimated total ESV and coefficient of sensitivity.

Change of value coefficient	1995		2005		2015		2025		2035	
	%	CS	%	CS	%	CS	%	CS	%	CS
Cropland VC ± 50%	14.04	0.28	15.83	0.32	16.18	0.32	16.56	0.33	16.90	0.34
Forestland VC ± 50%	0.83	0.02	0.83	0.02	0.83	0.02	0.82	0.02	0.82	0.02
Grassland VC ± 50%	28.45	0.57	27.31	0.55	27.40	0.55	27.49	0.55	27.56	0.55
Wetland VC ± 50%	1.03	0.02	1.42	0.03	1.79	0.04	1.06	0.02	1.08	0.02
Urban VC ± 50%	0.06	0.00	0.13	0.00	0.20	0.00	0.20	0.00	0.25	0.01
Bare land VC ± 50%	0.00	0.00	0.00	0.00	0.00	0.00	0.00	0.00	0.00	0.00
Waterbodies VC ± 50%	5.58	0.11	4.87	0.10	4.33	0.09	3.86	0.08	3.39	0.07

## Discussion

### Impact of LULC change on ecosystem services in Central Asia

Globally, patterns of LULC change are themselves changing rapidly due to the acceleration of processes such as population growth, expansion of urban areas, and agricultural intensification (Lambin & Geist, 2006), which could affect regional ecosystem services (Hu, Liu & Min, 2008; Nahuelhual et al., 2014); these effects are particularly prominent in the Central Asia region. The United Nations statistics show that Central Asia’s urban population increased by 37.97% between 1995 and 2015, from 24.01 million to 33.13 million, which led to the overexploitation of water and land resources to meet people’s needs for water, food, and energy (Granit et al., 2012). We found significant expansions of cropland (+22.10%) and urban areas (+322.40%) and shrinking of water bodies (−38.43%) and bare land (−9.42%) during 1995–2035 (Table 2). Correspondingly, cropland ecosystem services value increased by 93.45 billion US$ from 1995 to 2035, which was mainly caused by the expansion of cropland area (Table 3). However, the area of water bodies decreased sharply during 1995–2035, causing a loss of 64.38 billion US$ (Table 3).

Cropland changes may seem economically profitable, but a large increase in agricultural land can result in the loss of natural ecosystem services. More specifically, the impact of complex human agricultural activities on the ecosystem services of water quantity and quality is considerably overlooked by current agricultural management models that maximize food production and minimize other ecosystem services (Coupe, Barlow & Capel, 2012). We found that the expansion of agricultural land has largely led to an increase in the service functions of food production (+32.23 billion US$), raw materials (+32.23 billion US$), soil formation (+10.86 billion US$) and biodiversity (+9.72 billion US$) during 1995–2035 in Central Asia (Table 4). However, these findings are consistent with the results of numerous studies around the world showing that agriculture and urban expansion have a negative impact on the provision of other important ES, such as water regulation (−41.03 billion US$) (Schröter et al., 2005), gas regulation (−0.04 billion US$) (Wang et al., 2006), and recreation, cultural and tourism (−21.91 billion US$) (Nahuelhual et al., 2014). Especially in Amu River Basin, irrigation water consumption of crops such as wheat, cotton and maize increased by at least 60% from 1962 to 2002, leading to a significant reduction in the amount of water flowing into the Aral Sea (McDermid & Winter, 2017). Now the Aral Sea has shrunk to approximately 1/10 of its original area (Ablekim et al., 2017). Our results show that the ESV in Karakalpakistan, Uzbekistan, decreased by more than 50% during 1995–2035 (Fig. 4), which was mainly caused by the shrinkage of the Aral Sea area. Therefore, wider cultivation of crops with lower water consumption and higher added value would reduce the shrinkage of the Aral Sea and thereby enhance ecosystem services.

In addition, LULC changes are interrelated with other global processes such as climate change and land degradation (Kertész, Nagy & Balázs, 2019), which directly or indirectly affect local ecosystem services (Hossu et al., 2019; Qiao et al., 2019). For example, approximately 23% of farmland in the Amu Lowland has been degraded due to inappropriate land use activities (Djanibekov et al., 2018), resulting in the loss of biodiversity and the decline of carbon storage capacity, which will further affect agricultural production, disturbance regulation and climate regulation (Sutton et al., 2016). Namely, driven by common factors (e.g., land use changes and land degradation, etc.), synergies and trade-offs exist between system services between ESs over time (Cord et al., 2017).

### Limitations and areas of future research

Remote sensing images are the most important data source for research in ES, and LULC is the most widely used variable for assessment in ESV (Song, 2018). However, the limitations of global land cover data arise from the product generation process, including satellite sensor characteristics (spectral, temporal and spatial resolutions), definition and classification methods of land cover. Because of error propagation in the process of quantifying land cover data (Sexton et al., 2015), LULC change may be considerably underestimated or overestimated (Table 2). Such uncertainties are inevitably introduced in the analysis of spatial change patterns when the biomes used as proxies match LULC types (Kreuter et al., 2001). In this study, shrubs and sparse vegetation are used as grasslands (Table S1), which obviously exaggerates the area of grasslands and leads to an overestimation of ESV (Table 3). Therefore, to address these limitations, higher spatial resolution remote sensing data and more precise LULC classification will be used to further accurately assess ecosystem services in Central Asia.

Nine ecosystem service functions proposed by Xie et al. (2008) were selected to calculate the total ESV in Central Asia (Table 1), which could mask the value of other key ecosystem service functions. For example, sand fixation is an important function provided by desert ecosystems in arid and semi-arid areas, which has a close relationship with ecological benefits (Li & Xu, 2019; Musa, Deming & Cunyang, 2014; Taylor et al., 2017). Hao et al. (2013) found that the ESV of sand fixation in the Ulan Buh desert ecosystem was 0.68 billion US$ in 2009. However, there is no comprehensive assessment of ecosystem service functions for dryland ecosystem services in this study. To address this limitations, Schild et al. (2018) have identified and compiled a comprehensive database to estimate the ESV in arid areas. However, their findings indicate that there are still important limitations in the valuation of ESV in arid region. In further research, we will critically evaluate and further improve monetary valuation techniques to promote sustainable land management in Central Asia.

Our approach for estimating the ecosystem services value by using simple benefits transfer methods has certain limitations (Schmidt, Manceur & Seppelt, 2016). For example, we assumed homogeneity of ESV in the entire LULC category and generalized the unit values of one area as the average unit value of all areas. As Costanza et al. (2014) estimated, farmland ecosystem services provided $ 5567/ha in 2011 (Table 1). Although the average unit value provides a common value coefficient for all nations of the world to compare the relative differences, different types of farmland (e.g., rainfed cropland in northern Kazakhstan and irrigate cropland in Amu River Delta) have different crops planting structures and provide different ecosystem services and functions (Zheng, Zhang & Cao, 2018). In addition, the costs of ecosystem services (e.g., soil salinization, the loss of genetic resources, and water eutrophication) were ignored in assessing the value of ecosystem services, which might exaggerate the natural ecosystem services value (Cao et al., 2018). As Cao et al. (2018) revealed, the value of ecosystem services decreased by 52.66% in 2014 when the costs of water resources, investments in ecological protection, land rent and management were considered to estimate the net farmland ecosystem services value in China. In Central Asia, especially in Uzbekistan, irrigated agriculture remains the basis of the Uzbek economy, consuming more than 90% of the water resources and 228.40 kg/ha of fertilizers (FAO, 2015) and employing 33% of the labor force (UZSTAT, 2010). All of these factors suggest that farmland ecosystems have paid massive costs while providing a large number of services. However, because we did not incorporate these negative factors into the evaluation system, the ESV of farmland was overestimated (Table 3). Therefore, development of a framework that can assess the net value of ecosystem services is urgently needed to make reasonable arrangements for resource allocation.

We determined that the CS for the ESV was quite low (Table 5) when the value coefficients were adjusted by 50% to estimate the effects. However, we only focused on the directional change in ESV but ignored the magnitude of the ecosystem services values at specific points in time. It is worth noting that the interaction of some ES changed dramatically with different levels of economic development. For example, ESV of urban areas was overestimated (Table 3) in this study, masking the loss of essential ES provided by other LULC types. Yi et al. (2017) also believed that the value coefficient of urban built-up was assigned Costanza et al. (2014) seems unreasonable. We suggest that the value coefficients should be adjusted by collecting satellite or radar imagery and products obtained through efficient processing (e.g., soil moisture content, vegetation carbon stocks and chlorophyll content) to accurately reflect local ecosystem services of Central Asia in further studies.

## Conclusions

Our study showed that the increase in cropland ecosystem services value was approximately 67.89 billion US$ from 1995 to 2015, which was caused by the increasing areas (16.06%) of cropland. However, the area of water bodies decreased sharply by 38.34% during 1995–2035, causing a loss of 64.38 billion US$. It is important to note that cropland changes may seem economically profitable (e.g., food and raw material), but a large increase in agricultural land can result in the loss of natural ecosystem services (e.g., water regulation and climate regulation). Meanwhile, unsustainable agricultural practices and overgrazing practices are the major drivers of land degradation and desertification in the region. As a result, many ecosystem services are disappearing. In the future, we will make a precise assessment of ecosystem services in Central Asia by combining remote sensing observations and other technical means to understand the interaction of ecosystem services and manage the water-climate-food nexus to maximize its benefits.

##  Supplemental Information

10.7717/peerj.7665/supp-1 Table S1 LULC categories

10.7717/peerj.7665/supp-2 Table S2 The state names of a–r in Fig. 4

10.7717/peerj.7665/supp-3 Table S3 Markov chain matrix of LULCs transition probabilities for the period 1995–2005

10.7717/peerj.7665/supp-4 Table S4 Markov chain matrix of LULCs transition probabilities for the period 2005–2015

10.7717/peerj.7665/supp-5 Table S5 Markov chain matrix of LULCs transition probabilities for the period 1995–2015

10.7717/peerj.7665/supp-6 Figure S1 Value of ecosystem services (US$ ha year ^−1^) of per unit in Central Asia

## Additional Information and Declarations

### Data Availability

The following information was supplied regarding data availability: The raw data are available in the Supplemental Files, including the value coefficient proposed by Constanta et al. (2014) and the transfer matrix generated by CA-Markov Model. The raw transfer matrix data from IDRISI 17.0 is available in Table S3–S5. LULC categorical data is available in Table S1.
