# Supplementary material for: Variations in ecosystem service value in response to land use/land cover changes in Central Asia from 1995–2035
Source: PeerJ. 2019 Sep 12;7:e7665. doi: 10.7717/peerj.7665 (PMC6745190; doi:10.7717/peerj.7665)
Supplement: Table S1 [file peerj-07-7665-s001.docx]

| LULC | Value | Original LULC |
| --- | --- | --- |
| Cropland | 10 | Cropland, rainfed |
|  | 11 | Herbaceous cover |
|  | 12 | Tree or shrub cover |
|  | 20 | Cropland, irrigated or post-flooding |
|  | 30 | Mosaic cropland (>50%)/natural vegetation (tree, shrub, herbaceous cover) (<50%) |
|  | 40 | Mosaic natural vegetation (tree, shrub, herbaceous cover) (>50%)/cropland (<50%) |
| Forestland | 50 | Tree cover, broadleaved, evergreen, closed to open (>15%) |
|  | 60 | Tree cover, broadleaved, deciduous, closed to open (>15%) |
|  | 61 | Tree cover, broadleaved, deciduous, closed (>40%) |
|  | 62 | Tree cover, broadleaved, deciduous, open (15–40%) |
|  | 70 | Tree cover, needleleaved, evergreen, closed to open (>15%) |
|  | 71 | Tree cover, needleleaved, evergreen, closed (>40%) |
|  | 72 | Tree cover, needleleaved, evergreen, open (15–40%) |
|  | 80 | Tree cover, needleleaved, deciduous, closed to open (>15%) |
|  | 81 | Tree cover, needleleaved, deciduous, closed (>40%) |
|  | 82 | Tree cover, needleleaved, deciduous, open (15–40%) |
|  | 90 | Tree cover, mixed leaf type (broadleaved and needleleaved) |
|  | 100 | Mosaic tree and shrub (>50%)/herbaceous cover (<50%) |
|  | 110 | Mosaic herbaceous cover (>50%)/tree and shrub (<50%) |
| Grassland | 120 | Shrubland |
|  | 121 | Evergreen shrubland |
|  | 122 | Deciduous shrubland |
|  | 130 | Grassland |
|  | 140 | Lichens and mosses |
|  | 150 | Sparse vegetation (tree, shrub, herbaceous cover) (<15%) |
|  | 151 | Sparse tree (<15%) |
|  | 152 | Sparse shrub (<15%) |
|  | 153 | Sparse herbaceous cover (<15%) |
| Wetland | 160 | Tree cover, flooded, fresh or brakish water |
|  | 170 | Tree cover, flooded, saline water |
|  | 180 | Shrub or herbaceous cover, flooded, fresh/saline/brakish water |
| Urban | 190 | Urban areas |
| Bare land | 200 | Bare areas |
|  | 201 | Consolidated bare areas |
|  | 202 | Unconsolidated bare areas |
| Waterbodies | 210 | Water bodies |
