# Supplementary material for: Variations in ecosystem service value in response to land use/land cover changes in Central Asia from 1995–2035
Source: PeerJ. 2019 Sep 12;7:e7665. doi: 10.7717/peerj.7665 (PMC6745190; doi:10.7717/peerj.7665)
Supplement: Table S2 [file peerj-07-7665-s002.docx]

|  | Kyrgyzstan |  | Tajikistan: |  | Uzbekistan |
| --- | --- | --- | --- | --- | --- |
| a | Chüy | g | Sughd | k | Namangan |
| b | Ysyk-Köl | h | Rayons of Republican Subordination | l | Andijon |
| c | Naryn | i | Khatlon | m | Fergana |
| d | Osh | j | Gorno-Badakhshan | n | Sirdaryo |
| e | Jalalabad |  |  | o | Toshkent |
| f | Talas |  |  | p | Samarqand |
|  |  |  |  | q | Qasqadaryo |
|  |  |  |  | r | Surxondaryo |
