# Supplementary material for: Variations in ecosystem service value in response to land use/land cover changes in Central Asia from 1995–2035
Source: PeerJ. 2019 Sep 12;7:e7665. doi: 10.7717/peerj.7665 (PMC6745190; doi:10.7717/peerj.7665)
Supplement: Table S5 [file peerj-07-7665-s005.docx]

| LULC | Cropland | Forestland | Grassland | Wetland | Urban | Bare land | Waterbodies |
| --- | --- | --- | --- | --- | --- | --- | --- |
| Cropland | 0.88 | 0.02 | 0.07 | 0.00 | 0.03 | 0.00 | 0.00 |
| Forestland | 0.08 | 0.84 | 0.06 | 0.01 | 0.00 | 0.00 | 0.00 |
| Grassland | 0.19 | 0.00 | 0.80 | 0.00 | 0.00 | 0.01 | 0.00 |
| Wetland | 0.00 | 0.06 | 0.01 | 0.89 | 0.01 | 0.00 | 0.03 |
| Urban | 0.02 | 0.02 | 0.02 | 0.02 | 0.90 | 0.02 | 0.02 |
| Bare land | 0.01 | 0.00 | 0.15 | 0.00 | 0.00 | 0.83 | 0.00 |
| Waterbodies | 0.01 | 0.01 | 0.02 | 0.00 | 0.00 | 0.26 | 0.69 |
