# Supplementary figures and images for: Variations in ecosystem service value in response to land use/land cover changes in Central Asia from 1995–2035
Source: PeerJ. 2019 Sep 12;7:e7665. doi: 10.7717/peerj.7665 (PMC6745190; doi:10.7717/peerj.7665)

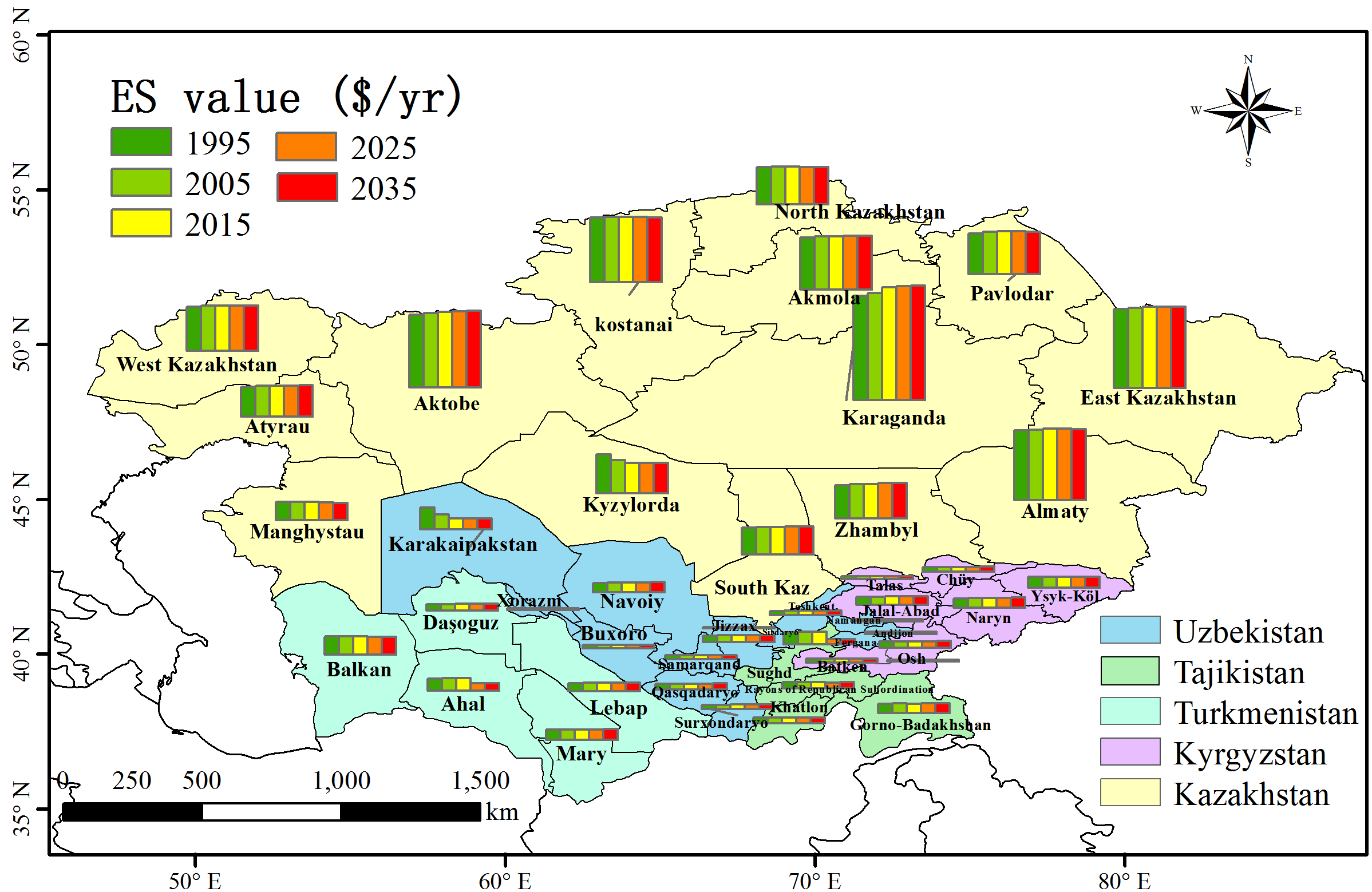

Supplement: Figure S1 [file peerj-07-7665-s006.png]
